# Concept for intrathecal delivery of brain recording and stimulation device

**DOI:** 10.3389/fmedt.2024.1211585

**Published:** 2024-02-08

**Authors:** Daniel P. Chapman, Jian-Young Wu

**Affiliations:** ^1^ Department of Neuroscience, Georgetown University, Washington, DC, United States; ^2^ Interdisciplinary Program in Neuroscience, Georgetown University, Washington, DC, United States

**Keywords:** interventional neurology, brain recording, medical device, ventricle anatomy, brain anatomy, brain-computer interface

## Abstract

Neurological disorders are common, yet many neurological diseases don't have efficacious treatments. The protected nature of the brain both anatomically and physiologically through the blood brain barrier (BBB) make it exceptionally hard to access. Recent advancements in interventional approaches, like the Stentrode™, have opened the possibility of using the cerebral vasculature as a highway for minimally invasive therapeutic delivery to the brain. Despite the immense success that the Stentrode™ has faced recently, it is limited to major cerebral vasculature and exists outside the BBB, making drug eluting configurations largely ineffective. The present study seeks to identify a separate anatomical pathway for therapeutic delivery to the deep brain using the ventricular system. The intrathecal route, in which drug pumps and spinal cord stimulators are delivered through a lumbar puncture, is a well-established route for delivering therapies to the spinal cord as high as C1. The present study identifies an extension of this anatomical pathway through the foramen of Magendie and into the brains ventricular system. To test this pathway, a narrow self-expanding electrical recording device was manufactured and its potential to navigate the ventricular system was assessed on human anatomical brain samples. While the results of this paper are largely preliminary and a substantial amount of safety and efficacy data is needed, this paper identifies an important anatomical pathway for delivery of therapeutic and diagnostics tools to the brain that is minimally invasive, can access limbic structures, and is within the BBB.

## Introduction

Neurological disorders including Alzheimer's disease, epilepsy, stroke, traumatic brain injury, Parkinson's disease, ALS, and multiple sclerosis caused 10 million deaths in 2019 and billions of dollars in healthcare spending, making neurological disorders the leading cause of disability in the world (1–3). Despite this tremendous health and economic burden placed on humanity by neurological disorders, many disease states remain with few or no effective treatments (ex: Alzheimer's and traumatic brain injury). Several challenges have limited the development of efficacious treatments for neurological disorders, a large portion of which are due to the anatomically and physiologically protected nature of the central nervous system. The blood brain barrier makes delivery of most biologic and pharmacological agents to the brain massively inefficient. Open brain surgery, during which part of the cranium is removed, remains a highly invasive and expensive operation even with the advent of less invasive techniques like keyhole surgery (4, 5). Thus, there is a need to develop novel techniques in which therapies can be delivered to the brain efficiently and in the outpatient setting.

**Figure 1:**
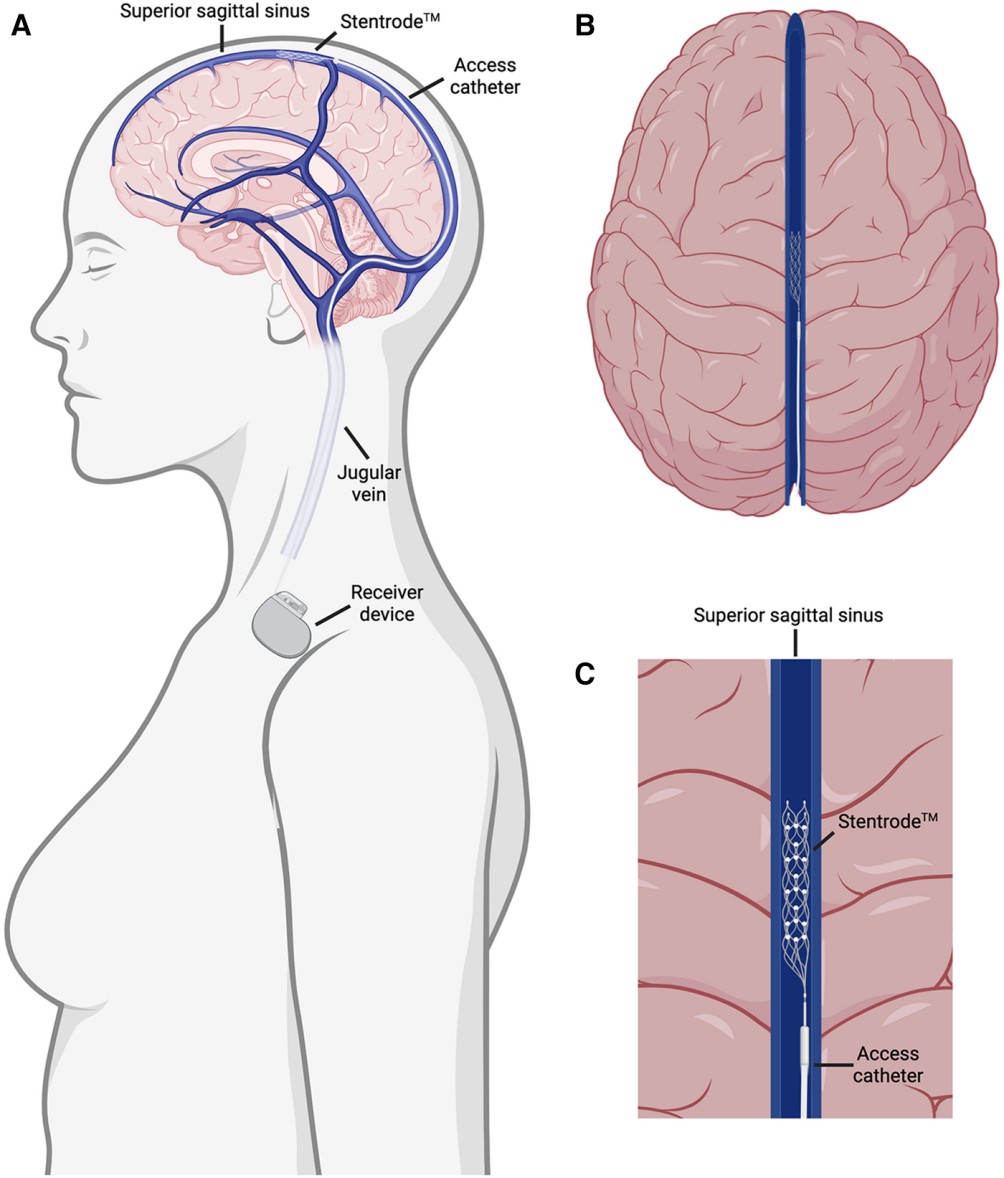
Overview of the Stentrode™ implantation. (**A**) The Stentrode™ is implanted through the jugular vein and into the superior sagittal sinus where it records electrical activity across the vascular surface. (**B**) Top down view of the Stentrode™ in the superior sagittal sinus. (**C**) Close up view of the Stentrode™ adjacent to brain parenchyma in the superior sagittal sinus.

Recently, an endovascular device called the Stentrode™, in which electrodes are attached to a stent scaffold, was developed and is close to commercialization (6–10). This device is implanted in the superior sagittal sinus through an interventional endovascular procedure and does not require surgery (Figures 1A,B). The resulting transvascular brain recordings are being used to facilitate a brain-computer interface for patients suffering from neuromuscular disorders (Figure 1C) (10). This represents a major stride forward in minimally invasive delivery of neurological therapies in the outpatient setting but is still faced with several major setbacks. Due to the vascular nature of this procedure, drug eluting versions of this device are ineffective as this technique remains outside the blood brain barrier. Furthermore, while the superior sagittal sinus is adjacent to the sensorimotor cortex, making it useful for facilitating brain-computer interfaces controlling motor actuators, the restriction of this device to the major sinuses on the external part of the cortex limits its scope to cortical applications (Figure 1C). Delivering drugs, electrical stimulation, and recording capabilities to deeper limbic structures that are inaccessible through the sinuses have shown promise in preclinical and clinical studies for treating epilepsy and memory disorders (11–20). Therefore, there is a need to effectively deliver therapeutics to limbic structures in the brain in a minimally invasive and safe manner.

While the Stentrode™ runs along the brains highways by snaking through the vascular system, devices such as the Ommaya Reservoir, which is implanted from the superior aspect of the cranium, have taken advantage of the brains sewage system by entering the ventricular system to house drug delivery (21, 22). Ventricular access routes in the spinal cord for treatment of chronic pain have become commonplace as well (23). Drug delivery cannulas implanted through a small needle into the epidural or subarachnoid space of the lumbar spine have accessed spinal nerves as high as C1 (24–26). A similar approach with foramen magnum or cervical puncture has been employed for spinal cord stimulators and drug pumps alike (27–29). Importantly, it has been noted that placement of cervical catheter tips do not increase the amount of complications when compared to placement of thoracic catheters (28). This suggests that navigating catheter tips through the cervical region can be done without substantial resistance from biological material and is safe in human patients.

Interestingly, the Foramen of Magendie is millimeters away from these cervical implants accessed through the lumbar spine in the subarachnoid space and provides a doorway into the upstream ventricular system which is adjacent to the brainstem and limbic areas of the brain. This pathway from the subarachnoid lumbar spine > Foramen of Magendie > fourth ventricle > the main ventricles is a potential access route to the brain that would provide direct access to limbic structures and bypasses the blood brain barrier if drug elution is desired. We sought to explore the feasibility of accessing different structures of the brain through an intrathecal ventricular access route with a novel self-expanding electrical stimulating and recording device. In the present study, we first explore the intrathecal ventricular access route, use data about ventricular anatomy to manufacture a device capable of navigating this route, and explore its potential in being implanted into the human brain in a minimally invasive fashion. This study is an important step forward in exploring new ways to deliver therapies to deep structures of the brain.

## Methods

### Device fabrication

We sought to create a device capable of fitting through the ventricular pathways of the brain. When navigating the ventricles, it is important to consider the diameter of each segment to prevent CSF blockage. The foramen of Magendie is the largest of the three apertures exiting the fourth ventricle and is commonly used as a surgical window for examining the floor of the fourth ventricle (30). The Sylvian aqueduct, which connects the third and fourth ventricle is extremely narrow and a common point of blockage (31). A review of previous literature suggests that this is the narrowest point in the ventricular system with the cross-sectional area ranging from 0.4 mm^2^ to 9.84 mm^2^ which correspond to a diameter of ∼0.7–3.5 mm although most adults fall between 1 and 3 mm diameter (31, 32). Thus, we sought to create a device capable of being implanted in the brains ventricular system with a diameter of less than 1 mm, although a device implanted on a living human would likely require <0.5 mm in diameter to preserve most of the cerebrospinal fluid flow. Thus, we manufactured devices of both 1.9 and 3.3 Fr. We focused on delivering electrodes to the brain capable of recording and stimulating neural activity across the ventricular membrane by designing a narrow-guided device with the capability to self-expand when *in situ*. This self-expansion property allows for the device to snake through the ventricles in its collapsed state and then expand to allow electrodes to be pressed against the wall of the ventricles increasing their spatial proximity to the desired brain area and increase recording quality.

**Figure 2:**
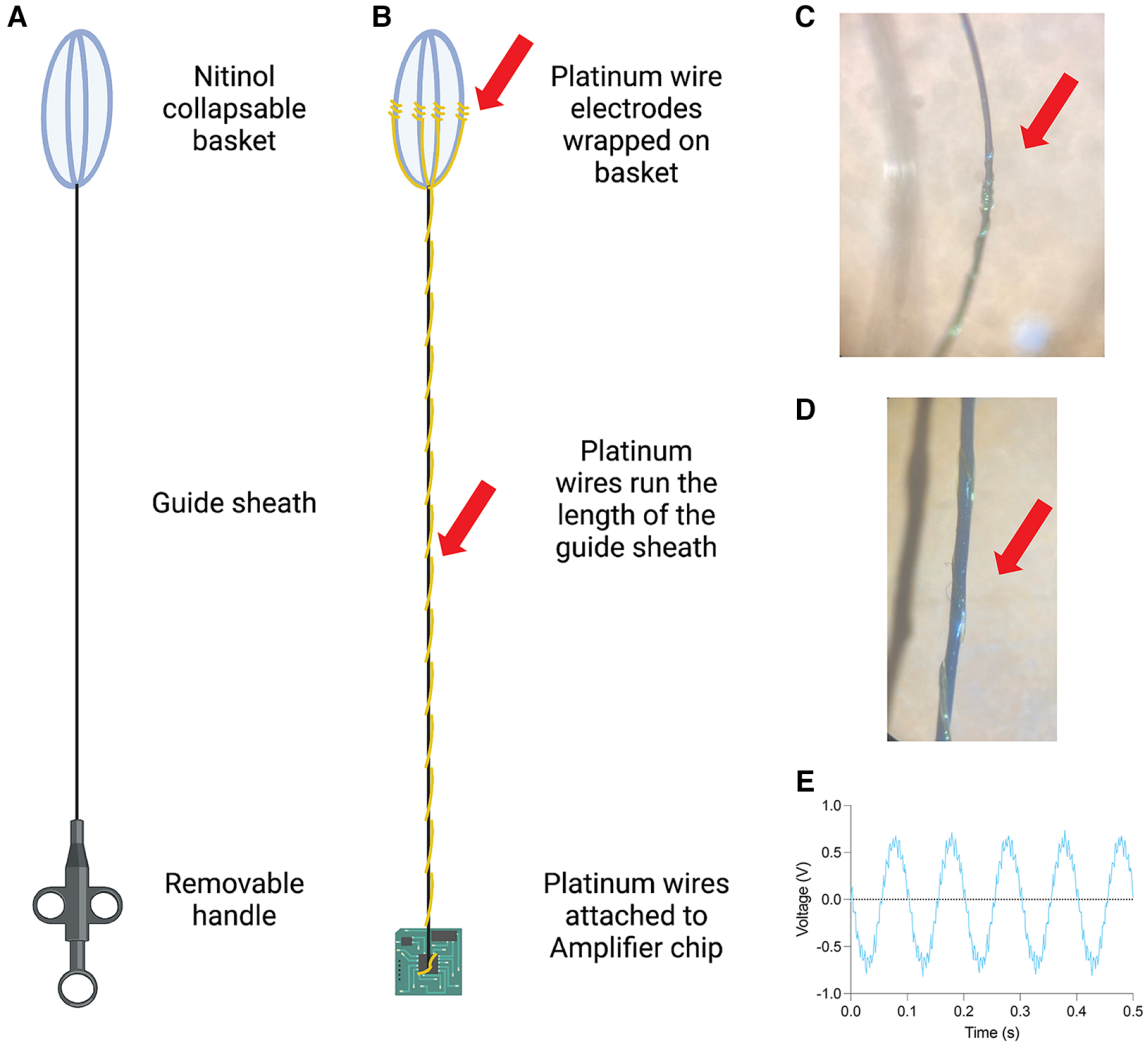
Overview of recording device that navigates ventricular system. (**A**) Schematic of Boston Scientific Zero Tip stone removal device. (**B**) Platinum wire electrodes were wrapped around the collapsible nitinol basket (top left) and run the length of the guide wire to a cup pin connector (bottom right) with schematic of the full device (right). (**C**) 2× view of platinum wire electrodes on collapsible sheath. (**D**) 2× view of platinum wire on length of guidewire. (**E**) Example recording of artificial 10 Hz oscillation from one of four platinum wire electrodes.

To fabricate a device capable of navigating the human ventricular system and expanding *in situ*, we attached 4 independent platinum wire electrodes to an expanding scaffold (Boston Scientific zero tip nitinol stone retrieval basket: Figure 2A) and fed both the scaffold and the electrode wires through a sheath that can advance and retract to collapse or expand the nitinol scaffold respectively. To do this, we removed the internal wire and self-expanding nitinol basket component of the stone retrieval device listed above. At the end of the device proximal to the nitinol scaffold, the electrically insulating coating on each of the platinum wires was removed using heat to allow for signal acquisition and the wires were tightly wrapped against the nitinol scaffold and adhered with glue (Figures 2B,C). We wire wrapped 4 independent PTFE coated platinum wires along the length of the wire (the linear component, not the nitinol basket) and adhered the wrap with a polyurethane coating (Figures 2B,D). At the end of the device distal to the nitinol scaffold, the free platinum wires were soldered to a cup pin connector which was subsequently wired to a signal acquisition device (Figure 2B). The recording capacity of this device was tested by placing it into a salt bath and generating an artificial 10 Hz signal (Figure 2E). The assembled platinum wire and nitinol scaffold device was fed back through the original sheath of the stone retrieval device so that it can be expanded and collapsed with the retraction and advancement of the sheath over the basket respectively (See Supplementary Movie 1).

The device described here was created with entirely off the shelf materials, but several alternative configurations exist. In general, the components are a long guidewire and expanding scaffold (here a stone retrieval basket was used); a guide catheter (here the sheath from the stone retrieval basket was used); electrodes attached to the scaffold and connected to the acquisition device (here platinum was used); and an acquisition component (here an Oscium mobile acquisition device was used). Any off the shelf existing devices could be used to make the device so long as the diameter of the scaffold and wire match the internal diameter of the guide catheter. Additionally, any electrophysiology acquisition device could be used at the distal end of the device that is attached to a cup pin connector. The self-expanding scaffold is nitinol in this case although any biocompatible shape memory alloy would suffice. The long guidewire of which the platinum electrode wires are wrapped around could be nitinol, stainless steel, titanium, or other flexible biocompatible metals used in guidewires. The sheath could be made of nitinol, stainless steel, titanium, nylon, platinum, polyester, or other flexible biocompatible materials commonly used in guide catheters. In the current prototype, the electrodes are platinum wire electrodes but other electrode materials such as gold, silver, or graphene would suffice. Electrodes could also be in disk or ball configurations. Importantly the recent use of polyimide in thin electrode sheets has shown a high degree of biological compatibility and good quality chronic recordings (33–37). Generating a polyimide electrode that could fold or roll up into the retractable sheath would be an excellent strategy for a future device as it would increase the biological compatibility and the number/density of electrodes that could be delivered in this way.

### Testing device

To test the recording capacity of the device, we placed it in a 5 ml salt solution (1 mM NaCl) at room temperature. An artificial alternating current of 10 Hz and 10 mA was generated in the salt solution. Recordings were acquired on an Oscium II mobile acquisition device. Recording files were exported from Oscium and plotted using Prism.

### Anatomical testing

To test if the device could fit in the ventricular system, a hemi-sected mid sagittal adult human brain section was used. The sample was cut at the level of the caudal brainstem. The septum pellucidum was removed to allow for visualization of the device *in situ*. For spinal cord simulation, two tubes 75 cm in length were used. The larger tube had an inner diameter of 2 cm and simulated the spinal canal while the smaller tube marked in red had an outer diameter of 1.3 cm and was fed through the bigger tube to simulate the spinal cord. This simulated spinal cord was continuous with the brainstem of the human brain sample at the level of the caudal spinal cord.

### Figures

All graphics in figures were created using BioRender.com.

## Results

### Proposed anatomical pathway from spine to ventricles

**Figure 3:**
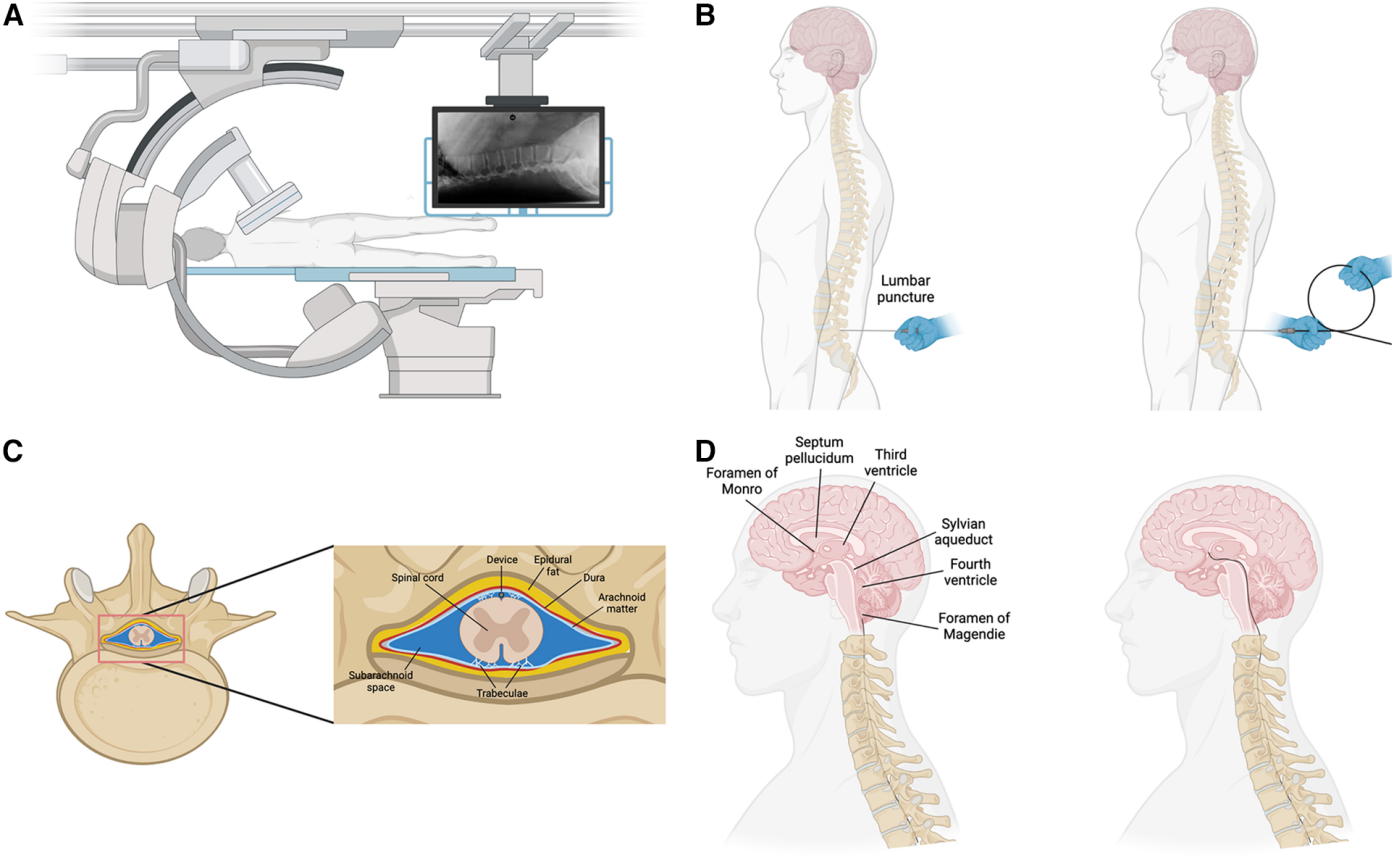
Overview of procedure to implant device. (**A**) Intraoperative imaging with c-arm x-ray machine. Patient in lateral recumbent on operating table. (**B**) Lumbar puncture into the subarachnoid space and advancement of the device into the spinal canal. (**C**) The device would be advanced up the spinal canal in the subarachnoid space on the dorsal side of the spinal cord as is down during implantation of drug pumps. (**D**) After reaching the brainstem, the device would enter the brain through the Foramen of Magendie, the fourth ventricle, and the Sylvian aqueduct.

Cerebrospinal fluid generated in the choroid plexus in the third and fourth ventricles, flows through the Sylvian aqueduct into the fourth ventricle and into the cisterna magna through the foramen of Magendie and Luschka on the medial and lateral aspects of the fourth ventricle respectively. The cisterna magna is continuous with the subarachnoid space of the spinal cord, which is accessible through lumbar puncture in the subarachnoid space. In this section, we highlight this anatomical series as a potential route for delivering drugs, devices, or other therapies to the ventricular system of the brain. We propose the following interventional endoventricular procedure to be done under intraoperative guidance with c-arm x-ray (Figure 3A). 1) lumbar puncture into the subarachnoid space with needle; replacement of puncture needle with access catheter; guide catheter is threaded up the subarachnoid space (Figure 3B) on the dorsal aspect of the spinal column (Figure 3C); guide device enters the fourth ventricle through the foramen of Magendie (Figure 3D); guide device traverses the fourth ventricle into the Sylvian aqueduct; guide device enters the third ventricle and optionally enters the lateral ventricles through the foramen of Monro; recording device is expanded into the desired brain structure through retraction guide device. This procedure would be done under local anesthetics and guided using intraoperative imaging such as x-ray fluoroscopy or computed tomography as is standard in placing drug pumps. A procedure such as the one described above is highly beneficial as it could complement open brain surgery, replace some operations, or open the door for entirely new procedures and therapies.

### Narrow self-expanding device fits through the ventricular system

**Figure 4:**
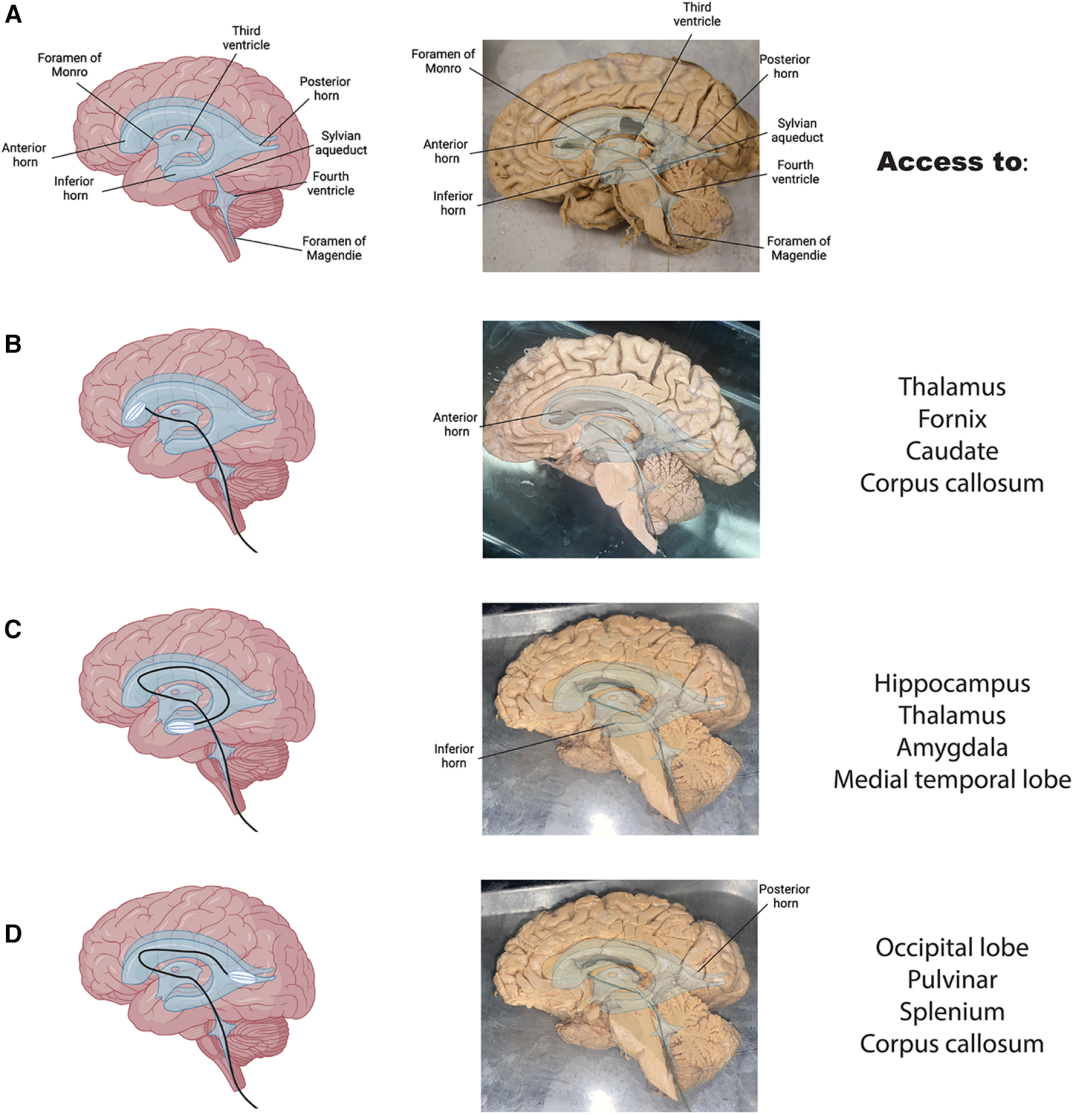
Access to clinically relevant brain regions. (**A**) Brain graphic with overlain ventricles in blue (left) and a hemisected human brain with overlain ventricles and labels (right). (**B**) The proposed route through the ventricular system to the anterior horn of the lateral ventricle in a graphic (left) and on hemisected brain (right) with anatomical landmarks adjacent to this structure listed on the far right. (**C,D**) Same as B for inferior (**C**) and posterior (**D**) horns respectively.

To test the feasibility of this anatomical pathway, we manufactured a recording device (see methods) capable of navigating the human ventricular system and expanding *in situ*. A schematic of a normal brain with overlain ventricles and an accompanying hemisected brain shows the path through the ventricular system (Figure 4A). We first tested the device through a simulated spinal cord and ventricular space on a human anatomical sample (Supplementary Movie 1). The device was successfully delivered intrathecally and entered the brain's ventricular system through the foramen of Magendie, fourth ventricle, cerebral aqueduct and into the third ventricle (Supplementary Movie 1).

To display the expanding nature of the device, we placed the device through the above pathway and into the anterior horn of the lateral ventricle in a sample with the septum pellucidum removed (Figure 4B). his would allow it to record and stimulate activity of adjacent neurons in a live subject. The collapsible nature of the device allows for an ease of passage through the ventricular system and into the desired target area. These findings suggest that the proposed anatomical pathway has the potential to be used for minimally invasive monitoring and treatment of various brain disorders. Pre-shaping of the device to match the individual brain samples was necessary to promote easy passage through the narrow ventricular pathways. This is common practice in other interventional procedures, especially when pre-operative imaging is available. Additionally, wetting the tissue increased the ease of passage of the device through the ventricles of the fixed tissue.

Delivery of drugs or electrical stimulation/recording could occur in any of the ventricles along the way. Fourth ventricle delivery would provide access to brainstem and cerebellar nuclei; third ventricle access would be optimal for delivery of therapeutics to the thalamus, hypothalamus, fornix, anterior commissure, lamina terminalis, optic chiasm, mamillary bodies, and tectum; lateral ventricle access would allow therapeutic delivery to the anterior horn, posterior horn, or ventral horn for various limbic structures including the thalamus, fornix, caudate, corpus callosum, hippocampus, amygdala, medial temporal lobe, occipital lobe, and splenium (31). All the structures mentioned above are only potentially accessible with the precision and degree of accessibility varying widely across different areas and the use case would dictate the desired brain target. Schematics showing the path through the ventricles and the device implanted in a hemisected human anatomical brain sample are depicted for each major structure of the lateral ventricles from two different brain samples (Figure 4B-4D). In general, third ventricle delivery could be useful for treatment resistant epilepsy, movement disorders (Parkinson's, essential tremor, etc.), and sleep disorders. Anterior horn of the lateral ventricle could be used to deliver therapies for movement disorders, mood disorders (depression, anxiety, and other mental health disorders), or epilepsy (Figure 4B). Posterior horn of the lateral ventricle would be targeted in disorders involving vision such as acquired blindness (Figure 4C). Inferior horn of the lateral ventricle could be targeted for memory disorders (Alzheimer's and other forms of dementia, traumatic brain injury, Wernicke-Korsakoff syndrome, etc.), epilepsy, mood disorders, and PTSD (Figure 4D). Intraventricular lesions such as neoplastic processes like choroid plexus papillomas could be accessed anywhere in the ventricles. Note, for the inferior and posterior horns, the expanded device cannot be visualized in a hemisected sample.

## Discussion

Here we explore the anatomical potential for intrathecal delivery of an electrode containing device to the ventricular system of the brain as a promising approach for the diagnosis and treatment of various brain disorders. One of the main advantages of this approach is its minimally invasive nature, as it avoids the need for invasive procedures such as craniotomy and piggybacks off well established protocols for spinal cord stimulation and spinal cord drug pumps (23–29). Furthermore, the ventricular system provides a direct route to the brain, allowing for the delivery of electrodes to specific regions of interest within the limbic system of the brain (31).

The current device has electrical stimulation and recording properties which are important for treatment and diagnosis/monitoring of neurological disease respectively. Brain stimulation functions by modulating neural activity through electrical current and deep brain stimulation currently has five FDA approved indications; Parkinson's Disease, dystonia, essential tremor, obsessive compulsive disorder, and epilepsy (38).

Electrical recordings can be done at multiple scales and have been employed for epilepsy monitoring and cortical mapping (39). Current limitations to stimulation/recording in the brain are largely related to delivery, especially to deep brain structures. Flexible recording arrays for electrocorticography have largely been used in basic research and epilepsy monitoring but require a craniotomy while deep brain stimulation probes are relatively safe to implant but limited to a very small cross-sectional area due to their passage through brain parenchyma during implantation (40). The approach described here would allow multichannel devices to be expanded within the lumen of the ventricle to allow for a broader surface area in stimulation/recording of deep brain structures without compromising for invasiveness.

Our first design of the device uses a nitinol basket as the expandable component of the array with platinum wire electrodes for recording/stimulation. This rudimentary design is stiff and, while compatible with short term implantation, is likely not suitable for chronic indications. Much work has been done on flexible, multichannel arrays with thin profiles in recent years (33–37). One major next step for this approach is to design an expandable flexible array that could be implanted in the ventricular wall to record high fidelity brain signals in the chronic setting.

The current report only presents a rough sketch of the general idea and a possible example design. We can envision that the actual design of the device would be quite different, e.g., using soft polymer scrips to replace platinum wire and using other mechanism to deploy the electrode net. These modifications would mostly occur after *in vivo* experiments in cadavers or large animal models. These experiments will be our future direction.

The internal diameter of the Sylvian aqueduct is the limiting factor of the diameter of the device. After implantation, the device left in the aqueduct should have insignificant impact to the maintain the CSF flow. We have described a small diameter of 1.9 Fr, which is ∼0.6 mm in the current design, but after implantation the thread left in the aqueduct might be further reduced to minimize irritation to the surrounding tissue. One possibility is to use a thin polyimide film thread to connect between the third and fourth ventricles. For example, a 100 um wide and 30 um thick polyimide film can be used to replace platinum wires in the current design. This would have insignificant impact to the CSF flow in the aqueduct. Polyimide is also more flexible than platinum, reducing the possibility of irritating the surrounding tissue in the aqueduct. The wires are made with gold plating on the polyimide surface, and four wires are sufficient to bring the multiplexed signals from hundreds of ventricular electrodes (41). One limitation to this approach and the device fabricated here is obtaining a good signal to noise ratio with a relatively long wiring distance between electrode and acquisition device (∼1 meter). This remains a challenge in obtaining clinically relative recordings from brain tissue producing uA worth of current.

While intrathecal delivery of a device to the ventricular system has shown promise in this anatomical study, there are still several challenges that need to be addressed. These include the need for improved targeting and delivery methods to ensure precise and effective delivery of therapeutics to specific regions of interest within the brain in a safe manner. Intraoperative imaging techniques such as x-ray fluoroscopy, CT, or MRI are well established in other interventional procedures (6–10, 42), and it is likely that implementation of this pathway would rely on one or more imaging modalities. With regards to electrical recording and stimulation specifically, it is also unknown how the quality of transventricular recordings would be compared to other electrode locations. It is reasonable to assume that transventricular recordings are possible and reliable as direct comparisons of endovascular recordings to subdural and epidural signals show little to no loss of signal (8).

Additionally, the safety and efficacy of these approaches need to be carefully evaluated before clinical studies can be considered. Several complications can arise from intrathecal drug therapy such as CSF leakage, infection, bleeding, seroma, and various neurological injury (23) and it is likely that these complications would also arise in intrathecal delivery to the brain due to the same lumbar puncture initiating the procedure. As mentioned above, intrathecal delivery of shunts may prove as a useful tool in treating hydrocephalus, however, intrathecal device delivery may pose a risk for causing hydrocephalus as well. The brains ventricular system is unique compared to the spinal column as it narrows substantially in communicating foramen and the Sylvian Aqueduct to less than a centimeter in diameter in some patients. Ensuring appropriate CSF flow likely using contrast imaging is paramount to demonstrating the safety and efficacy of the intrathecal delivery technique. Next steps to address this would be to mathematically model CSF flow through the brain with different diameters obstructing the various foramen and subsequent CSF flow measurements implanted in an *in vivo model*. It is likely that the curvature of the device *in situ* would have complex effects on CSF flow, thus the diameter and shape of the device must be optimized to ensure with absolute certainty that CSF flow would not be obstructed. The current design of the device is a linear conformation through the spine and ventricular system. It is also unclear how the physiological bending of the spine, particularly in the cervical region, would affect the stability of the device. Further work is needed to model how anterior and lateral motion of the neck would disrupt device positioning in a live subject. This is both important for the positioning of the device at a desired target and safety concerns that may arise from traction between the device and local anatomical structures.

An additional potential complication would be perforation of the ventricular wall by the device and subsequent damage to the surrounding brain tissue. The current study uses only fixed tissue and non-biological anatomical models which is not representative of the fragility of live biological tissue. Overall, blood vessel puncture in interventional procedures is very low (43) and it would be desirable to achieve similarly low numbers of ventricular perforation as the complications would be equally as burdensome.

Other future directions include generalizing the anatomical approach to other devices such as drug pumps, shunts, and CSF sampling devices could be delivered intrathecally and into the ventricular system to diagnose and treat various neurological disorders. Since the ventricular system is within the blood brain barrier, targeted drug delivery using a similar device manufactured in conjunction with off the shelf drug pumps could be made. Additionally, sampling the cerebral spinal fluid at targeted areas in the brains ventricular system could also be performed in a diagnostic configuration of the device and delivery route.

In conclusion, the delivery of an intrathecal device to the ventricular system of the brain is a promising approach for the diagnosis and treatment of various brain disorders. Further research is needed to fully explore the potential of this approach, and to address the challenges associated with its use in clinical settings. With continued advancements in drug delivery and device technology, this approach has the potential to revolutionize the diagnosis and treatment of brain disorders as an outpatient procedure in the future.

## Data Availability

The raw data supporting the conclusions of this article will be made available by the authors, without undue reservation.

## Supplementary material

Supplementary Video 1 Simulated intrathecal delivery.
